# A Rare Case of Isolated Abdominal Aortic Injury in Blunt Abdominal Trauma: A Case Report

**DOI:** 10.7759/cureus.92638

**Published:** 2025-09-18

**Authors:** Prajwal Dhakal, Pradeep R Regmi, Shashi S Shingh, Binaya Adhikari, Saubhagya Dhakal

**Affiliations:** 1 Radiology, Institute of Medicine, Maharajgunj Medical Campus, Kathmandu, NPL; 2 Radiology, Tribhuvan University Institute of Medicine, Kathmandu, NPL; 3 Radiology, University of Iowa Hospitals and Clinics, Iowa, USA

**Keywords:** aortic injury, blunt abdominal trauma, contrast-enhanced computed tomography, isolated, management

## Abstract

Traumatic abdominal aortic injury (TAAI) rarely occurs in isolation and is usually associated with spinal, solid organ, or other hollow viscus injuries. Contrast-enhanced computed tomography (CECT) is the preferred modality of investigation. CECT can provide accurate details about the grade, location, and extent of injury. These details are essential in planning the management. Depending upon the grade of injury, management can be conservative or surgical. Low-grade injuries can be managed conservatively. Here, we present a case of a 26-year-old male patient with a low-grade isolated abdominal aortic injury diagnosed on CECT following blunt abdominal trauma, with an initial negative focused assessment with sonography in trauma (FAST) scan and persistent abdominal pain, managed successfully with conservative treatment.

## Introduction

Traumatic abdominal aortic injury (TAAI) is an infrequent injury, occurring in 0.08%-0.62% of cases of blunt abdominal trauma and representing 4%-6% of total aortic injuries [1]. Though uncommon, it can be potentially fatal [1,2]. Traumatic aortic injuries commonly involve the isthmus and descending thoracic aorta. Isolated abdominal aortic injuries are rarely reported. Abdominal aortic injuries, when they occur, are usually associated with other solid organs, hollow viscus, or spinal injuries [1-3]. Here, we present a case of a 26-year-old male patient who presented with abdominal pain following a motor vehicle accident, underwent a contrast-enhanced computed tomography (CECT) scan, was diagnosed with isolated low-grade aortic injury, and was managed conservatively.

## Case presentation

**Figure 1:**
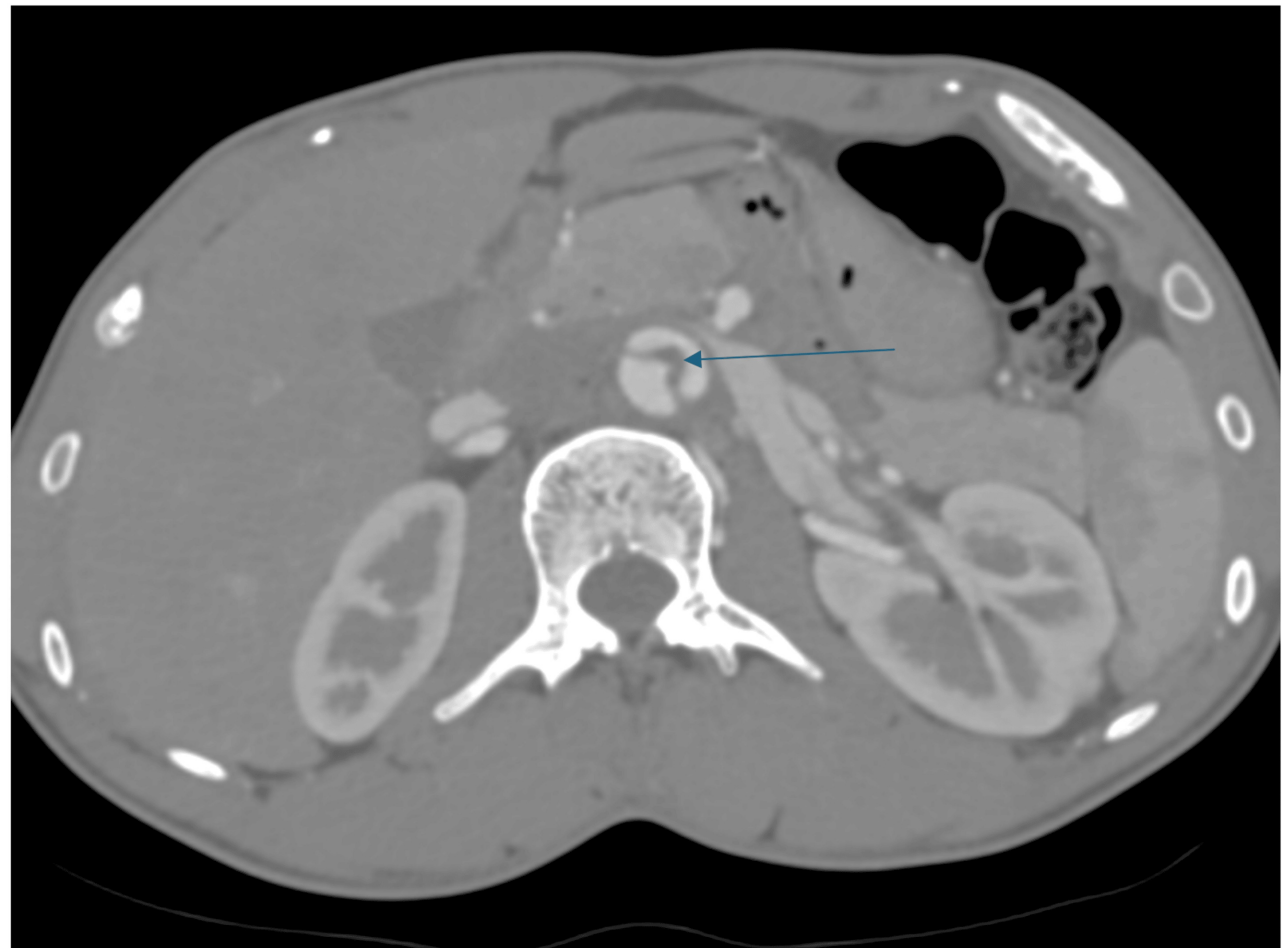
CECT abdomen axial section showing hypodense dissection flap (shown by thin arrow) within the lumen of the abdominal aorta CECT: contrast-enhanced computed tomography

A 26-year-old man presented to the emergency department following a motor vehicle accident with blunt impact to the abdomen. No obvious external injuries were present. The patient, however, complained of severe abdominal pain. The focused assessment with sonography in trauma (FAST) scan was negative with no obvious intra-abdominal or pelvic collection. Due to the persistence of abdominal pain, a CECT of the abdomen and pelvis was done. CECT showed an intraluminal hypodense linear flap extending just below the level of the left renal artery, dividing the lumen into two halves, i.e., true and false lumen (Figure 1).

**Figure 2:**
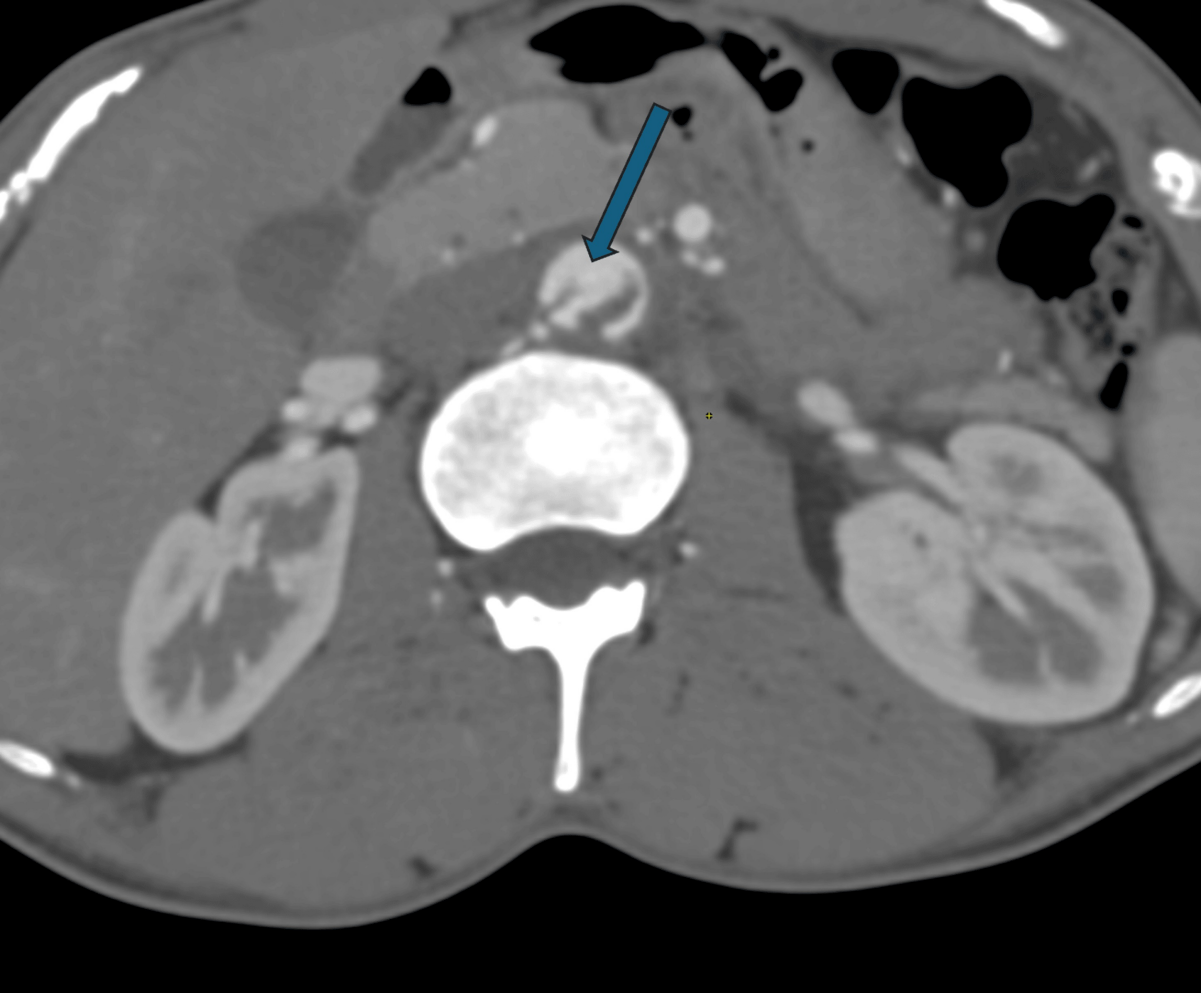
CECT abdomen axial section showing discontinuity in the dissection flap (shown by thick arrow) CECT: contrast-enhanced computed tomography

Discontinuity was, however, seen in the flap inferiorly, suggesting a patent communication between the true and false lumen (Figure 2).

**Figure 3:**
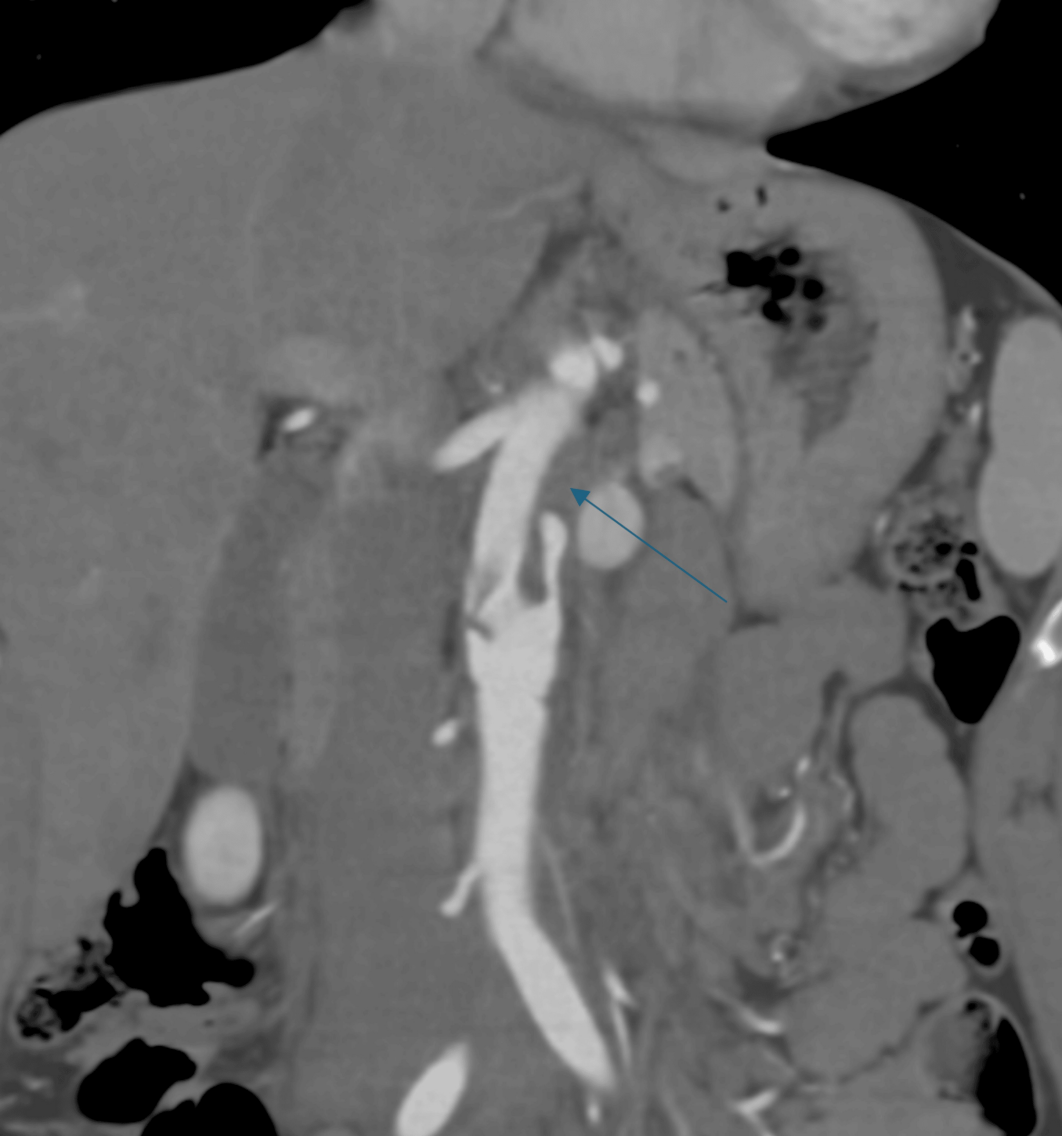
CECT abdomen coronal section showing hypodense filling defect, suggestive of thrombus (shown by thin arrow) within the proximal part of the false lumen CECT: contrast-enhanced computed tomography

A hypodense filling defect (thrombus) was seen in the proximal part of the false lumen (Figure 3).

The patient was diagnosed with a Grade I aortic injury. No other associated injuries were noted. No evidence of intramural hematoma, retroperitoneal collection, pseudoaneurysm, or rupture was found. The patient was managed conservatively. He was monitored for hemodynamic stability as well as with regular bedside ultrasound screening of the abdomen and pelvis to rule out the development of any retroperitoneal collection. The patient got symptomatically better with the resolution of abdominal pain, was hemodynamically stable throughout the hospital stay, and was discharged on the seventh day.

## Discussion

TAAI is usually caused by penetrating rather than blunt trauma [2]. In blunt trauma, it rarely occurs in isolation and is usually associated with solid organ injury, bowel or mesenteric injury, hemoperitoneum, or spine injury [1,3]. Also, the occurrence of aortic intimal tear or dissection is infrequent compared to aortic rupture. However, there is inadequate information on the frequency of these conditions. This may be due to the high mortality associated with these conditions before patients reach a healthcare facility, making accurate diagnosis difficult [3]. Society of Vascular Surgery (SVS) grades aortic injury as Grade I (showing intimal flap), Grade II (with intramural hematoma), Grade III (pseudoaneurysm), and Grade IV (rupture). Grade I and Grade II are considered to be less severe injuries, while Grade III and IV injuries are considered to be more severe injuries. Clinically stable patients with Grade I as well as Grade II injuries can be managed conservatively. Higher-grade injuries usually require surgical or endovascular management [4]. Studies, however, show that even the cases with severity up to small pseudoaneurysms showed a positive outcome with conservative management [5].

CECT angiography of the abdomen is considered to be the gold standard investigation for suspected abdominal aortic injury [3]. A CT scan can demonstrate direct signs of injury, including intimal flap, intramural hematoma, focal thrombus, contrast extravasation, pseudoaneurysm, or focal contour abnormality. It can also demonstrate indirect signs of injury like retroperitoneal hematoma and fat stranding [4]. Its accuracy in detecting the intimal flap of dissection is 100% [3]. A CT scan can also aid in diagnosing other associated solid organs, as well as bowel injuries if associated [4]. 

On the basis of treatment options for aortic injuries, the abdominal aorta is divided into three zones. Zone I extends from the diaphragmatic hiatus to the superior mesenteric artery (SMA). Zone II extends from the SMA to the renal arteries. Zone III extends from the renal arteries to the aortic bifurcation. Zone I and III injuries can usually be managed with both open and endovascular treatment. Zone II injuries are challenging to manage due to difficulty in open surgical management as well as lack of availability of endovascular devices in acute urgent settings [1]. Vascular surgeons need proper information about the location, type, and extent of injury to plan further steps in the management of TAAI, which can be done with the help of a CT scan. A CT scan is also helpful in proper follow-up of the condition, whether managed conservatively or with endovascular as well as open surgical approaches [6].

Ultrasound can also be used as a valuable screening tool to detect intimal tears and dissection, as studies have shown it to have a sensitivity of 67%-80% and a specificity of 99%-100% in detecting intimal flaps [7,8]. It could be a valuable screening bedside tool in monitoring the status of flaps. However, in an acute trauma setting, FAST scan is used as the preferred screening modality of investigation, which focuses on intraperitoneal fluid collection and does not include dedicated aortic as well as other solid organ evaluation. A retrospective study done by Natarajan et al. in 2980 trauma cases showed FAST scan to be effective tool for screening in hemodynamically unstable patients to guide further management, whereas it was considered to be ineffective in hemodynamically stable patient in detecting solid organ, hollow viscus as well as vascular injuries, where the sensitivity of FAST scan was found to be 41%, compared to overall sensitivity of between 63% and 96% in literature [9]. In our case, no intrabdominal fluid collection was seen, the patient was hemodynamically stable; however, due to the persistence of abdominal pain, a CT scan was done, and a diagnosis could be made despite negative FAST scan results.

## Conclusions

Isolated abdominal aortic injuries are a rare form of injury that can occur with blunt abdominal trauma. In hemodynamically stable patients and when not associated with other injuries or intrabdominal collection, it can be overlooked in a FAST scan. Information about the location, extent, and grade of injury is crucial in guiding the management. CECT, preferably, CT angiography, is the gold standard investigation which providesan accurate description of the injury necessary in guiding the management.
